# Diagnostic accuracy of intraoperative pelvic autonomic nerve monitoring during rectal surgery: a systematic review

**DOI:** 10.1007/s10151-024-03043-w

**Published:** 2024-12-06

**Authors:** A. O’Connor, C. Rengifo, B. Griffiths, J. A. Cornish, J. P. Tiernan, Jim Khan, J. W. Nunoo-Mensah, K. Telford, D. Harji

**Affiliations:** 1 Department of Colorectal Surgery, Manchester University NHS Foundation Trust, Manchester, UK; 2 Faculty of Biology, Medicine and Health, The University of Manchester, Oxford Road, Manchester, M13 9PL UK; 3 Department of General Surgery, Cardiff and Vale University Health Board, Cardiff, UK; 4 John Goligher Colorectal Unit, St James’s University Hospital, Leeds, UK; 5 Department of Colorectal Surgery, Portsmouth Hospitals NHS Trust, Portsmouth, UK; 6 Department of Colorectal Surgery, King’s College Hospital Foundation NHS Trust, London, UK

**Keywords:** Colorectal surgery, Neuromonitoring, Total mesorectal excision, Faecal incontinence, Urinary dysfunction, Sexual dysfunction

## Abstract

**Purpose:**

Anorectal and urogenital dysfunctions are common after rectal surgery and have a significant impact on quality of life. Intraoperative pelvic autonomic nerve monitoring (pIONM) has been proposed as a tool to identify patients at risk of these functional sequelae. This systematic review aims to evaluate the diagnostic accuracy of pIONM in detecting anorectal and urogenital dysfunction following rectal surgery.

**Methods:**

A systematic review of articles published since 1990 was conducted using MEDLINE, Embase, CINAHL, Google Scholar, Scopus, and Web of Science. Studies describing pIONM for rectal surgery and reporting anorectal or urogenital functional outcomes were included. The risk of bias was assessed using the QUADS-2 tool. The diagnostic accuracy of pIONM was established with pooled sensitivity and specificity alongside summary receiver-operating characteristic curves.

**Results:**

Twenty studies including 686 patients undergoing pIONM were identified, with seven of these studies including a control group. There was heterogeneity in the pIONM technique and reported outcome measures used. Results from five studies indicate pIONM may be able to predict postoperative anorectal (sensitivity 1.00 [95% CI 0.03–1.00], specificity 0.98 [0.91–0.99]) and urinary (sensitivity 1.00 [95% CI 0.03–1.00], specificity 0.99 [0.92–0.99]) dysfunction.

**Conclusions:**

This review identifies the diagnostic accuracy of pIONM in detecting postoperative anorectal and urogenital dysfunction following rectal surgery. Further research is necessary before pIONM can be routinely used in clinical practice.

**PROSPERO Registration Details:**

CRD42022313934.

## Introduction

Total mesorectal excision (TME) has improved oncological outcomes in rectal cancer [1]; however, a substantial risk of postoperative functional complications remains. It is estimated that anorectal dysfunction (AD) occurs in 50 to 90% of patients, with symptoms of faecal incontinence, frequency, urgency and stool clustering commonly described as ‘low anterior resection syndrome’ (LARS), which has a significant impact on quality of life (QoL) [2, 3]. Additionally, urinary dysfunction (UD) and sexual dysfunction (SD) have been reported in up to 30 and 50% of patients, respectively [4, 5]. Functional consequences have also been reported in rectal surgery for benign disease, with anorectal dysfunction ranging from 11 to 33% and urogenital dysfunction ranging from 16 to 22% in patients undergoing rectopexy with posterior mobilisation [6–9].

There are a range of risk factors for pelvic nerve damage including neoadjuvant radiotherapy, tumour characteristics and neuroanatomical variations; however intraoperative nerve injury is considered the principal cause [10–14]. Improved visualisation of pelvic nerves using minimally invasive platforms, including laparoscopic and robotic surgery, has been associated with improved functional outcomes [15, 16]. However, a visually preserved nerve does not necessarily confer functional integrity [17, 18]. Pelvic intraoperative nerve monitoring (pIONM) has been proposed as a tool during minimally invasive or open surgery to identify pelvic autonomic nerve injury [19]. Intraoperative nerve monitoring is used in other surgical specialities, including spinal and thyroid surgery [20, 21]. Nonetheless, limited data exist regarding its use in rectal surgery, including its diagnostic accuracy in identifying postoperative functional complications.

This systematic review aims to assess the diagnostic accuracy of pIONM in the identification of postoperative anorectal and urogenital dysfunction in patients undergoing rectal surgery.

## Materials and methods

### Study protocol

This systematic review has been reported according to the 2020 Preferred Reporting Items for Systematic Reviews and Meta-Analysis (PRISMA 2020) guidelines[22]. Details of the protocol were registered prospectively on the international prospective register of systematic reviews database (PROSPERO):

www.crd.york.ac.uk/PROSPERO/display_record.asp?ID=CRD42022313934.

The primary outcome was the diagnostic accuracy of pIONM in the identification of postoperative anorectal or urogenital dysfunction after rectal surgery. The diagnosis of these functional complications relies on validated patient-reported symptom severity measures.

Secondary outcomes include the techniques of pIONM, patient-reported outcome measures used, postoperative functional results in patients undergoing pIONM, and of any reported controls, and any reported adverse events attributed to pIONM.

### Inclusion and exclusion criteria

All studies were included which reported postoperative anorectal, urinary or sexual outcome measures in adult human participants undergoing surgery (open, laparoscopic or robotic) involving rectal mobilisation for benign or malignant conditions with pIONM. Studies with a control group were also included. There was no language restriction on included studies. Conference abstracts, case reports, letters to the editor and studies with no reported outcome measures were excluded.

### Literature search

A search was performed in November 2023 using MEDLINE, Embase, CINAHL, Google Scholar, Scopus and Web of Science for articles published between January 1990 and November 2023 using the search strategy: ((intraoperative and monitor*) or (nerve* and monitor*) or neuromonitor* or neuro monitor*) AND (rectal or rectum or total mesorectal or TME). Reference lists of included articles were screened for further reports.

### Study selection and data extraction

Following the literature search, duplicates were removed, and titles and abstracts screened with remaining records submitted for full-text review. Literature screening and data extraction were performed by two independent blinded researchers (AOC and CR). Discrepancies were resolved by mutual discussion or, where this did not result in agreement, by a third researcher (DH).

Studies which reported the same pIONM technique and outcome measure were grouped for data syntheses to establish the primary outcome.

### Risk of bias assessment

The risk of bias was assessed using the The Cochrane Collaboration-recommended QUADS-2 tool for diagnostic accuracy studies [23] by two independent blinded researchers (AOC & CR).

### Data analysis

Data analysis was performed using RevMan version 5.4.1 (The Cochrane Collaboration, 2020) [24], and MetaDTA v.2.01 (https://crsu.shinyapps.io/dta_ma/) [25] was used to calculate pooled sensitivity and specificity. Summary receiver-operator curves (SROC) were generated using the Moses-Littenberg model [26].

## Results

**Fig. 1:**
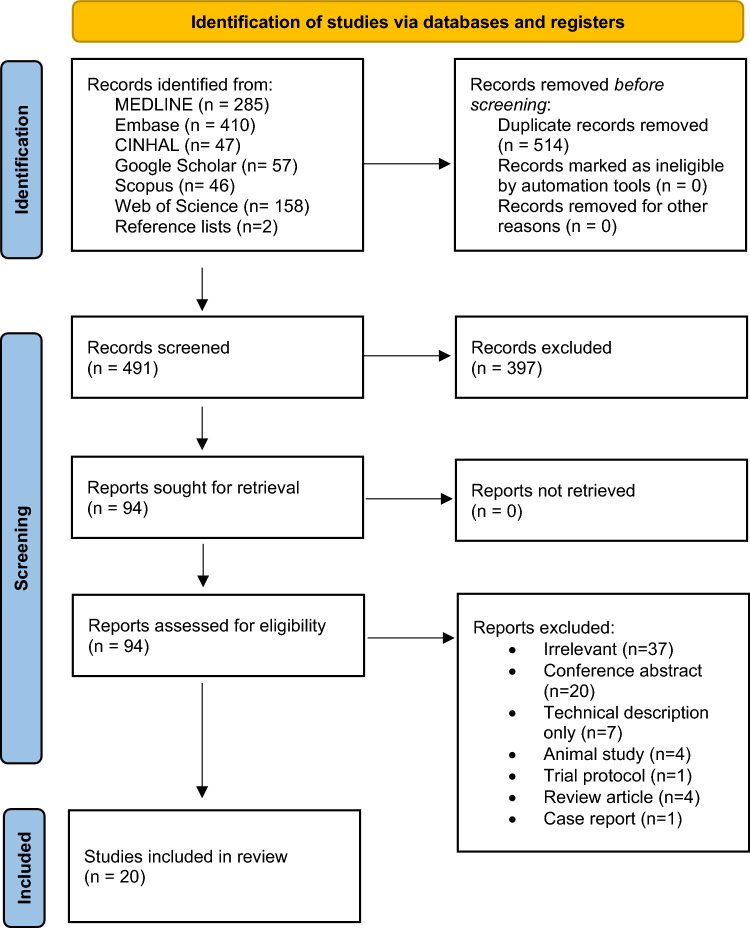
PRISMA flow diagram

A total of 1005 records were retrieved, which contained 491 unique results. After abstracts and titles were screened, 94 studies underwent full-text review. A further 74 records were removed, leaving 20 studies in this review (Fig. 1) [17, 18, 27–44].

Study characteristics, patient demographics and surgical data are displayed in Table 1. Eighteen prospective [17, 18, 28, 30–44] and two retrospective [27, 29] studies involving 686 patients were included, with seven studies reporting a control group [17, 27–30, 33, 44]. Only one randomised controlled trial was included comparing pIONM to control in patients undergoing TME (Trial of NEUROmonitoring System [NEUROS] study) [44]. Only two studies reported rectal surgery for benign conditions, with resection rectopexy (*n* = 12) being the most frequent procedure [32, 35]. The remaining 18 studies reported results from rectal cancer surgery.

**Table 1:** Study characteristics

Year of publication	Author	Study period	Type of study	Groups (*n*)	Sample (*n*)	Age median (IQR)	Gender (males/females)	Follow-up (months) median (range)	Operation (*n*)	Robotic/laparoscopic/open	Neoadjuvant treatment (*n*)
2022	Kneist et al. [44]	Jun 2012–Dec 2018	Prospective	pIONM	82	Mean 61 (± 11.4)	49/33	12	LAR (78) APR (4)	3/66/13	CRT (42) RT (3) CT (4) Unkown (1)
				Control	89	Mean 63.7 (± 9.7)	57/32		LAR (85) APR (4)	5/71/13	CRT (43) RT (4) CT (8) Unknown (1)
2020	Jin et al. [27]	Jan 2012–Dec 2018	Retrospective	pIONM	43	Bilateral pIONM: Male: 56 (38–64) Female: 54 (48–60) Unilateral pIONM: Male: 55 (42–65) Female: 53 (47–60)	29/14	12	LAR (35) APR (5) ISR (3)	0/43/0	NR
				Control	36	Male: 55 (36–64) Female: 52 (33–60)	23/13		LAR (29) APR (4) ISR (3)	0/36/0	NR
2020	Kauff et al. *[28]	Jan 2008–Oct 2015	Prospective	pIONM	29	63 (55–74)	26/3	24	LAR (29)	0/5/24	CRT (12)
				Control	23	64 (58–73)	12/11		LAR (23)	0/2/21	CRT (6)
2019	Zhou et al. [29]	Jan 2012–May 2016	Retrospective	pIONM	58	65 (45–80)	36/22	12	LAR (58)	0/58/0	CRT (19)
				Control	29	62 (55–78)	17/12		LAR (29)	0/29/0	CRT (7)
2017	Kauff et al. *[30]	Jan 2008–Aug 2014	Prospective	pIONM	43	65 (54–74)	35/8	24	LAR (32) APR (11)	0/11/32	CRT (16)
				Control	42	66 (57–75)	24/18		LAR (30) APR (12)	0/10/32	CRT (16)
2016	Kneist et al. *[42]	May 2013–Feb 2015	Prospective	pIONM	10	57 (51–61)	9/1	15 (6–20)	taTME (10)	0/10/0	CRT (7) CT (1)
2016	Kauff et al. *[18]	Feb 2008–May 2015	Prospective	pIONM	30	62 (51–71)	21/9	9 (6–12)	LAR (30)	0/30/0	NR
2015	Fang et al. [17]	Sep 2013–Sep 2014	Prospective	pIONM	71	Mean 58.0 (± 11.0)	45/26	6	LAR (63) APR (8)	0/71/0	CRT (19)
				Control	118	Mean 57.4 (± 11.7)	74/44		LAR (96) APR (22)	0/118/0	CRT (38)
2014a	Kneist et al. *[31]	NR	Prospective	pIONM	17	60 (54–73)	17/0	9 (8–12)	LAR (17)	0/0/17	CRT (9)
2014b	Kneist et al. *[32]	NR	Prospective	pIONM	10	51 (range 24–71)	7/2	6	PST (2) RPC (2) RR (2) LAR (2) APR (2)	0/10/0	RT (2)
2013a	Kneist et al. *[33]	Jan 2008—Nov 2012	Prospective	pIONM	15	65 (range 50–83)	13/2	10 (7–20)	LAR (15)	0/0/15	CRT (6)
				Control	15	66 (range 50–77)	13/2		LAR (15)	0/0/15	CRT (6)
2013	Kauff et al. *[34]	NR	Prospective	pIONM	35	5 (56–73)	31/4	6	LAR (35)	NR	RT (13) CRT (1)
2013b	Kneist et al. *[35]	NR	Prospective	pIONM	10	55 (range 38–75)	0/10	3	RR (10)	0/10/0	N/A
2012	Kneist et al. *[36]	NR	Prospective	pIONM	14	67 (range 50–89)	11/3	6 (2–12)	LAR (11) APR (3)	NR	CRT (6) CT (1)
2007a	Kneist et al. * ^ [37]	Apr 2002–Oct 2005	Prospective	pIONM	62	65 (range 29–84)	43/19	17 days (8–46)	PME (4) LAR (47) APR (11)	NR	CRT (7)
2007b	Kneist et al. * ^ [38]	Apr 2002–Oct 2005	Prospective	pIONM	62	65 (range 29–84)	43/19	20 (3–40)	PME (4) LAR (47) APR (11)	NR	CRT (7)
2007c	Kneist et al. *[39]	May 2002–Apr 2006	Prospective	pIONM	26	63 (range 29–70)	26/0	23 (4–41)	LAR (19) APR (5) PC (2)	NR	RT (5) CRT (3)
2005	Kneist et al. *[40]	Apr 2002–Jun 2003	Prospective	pIONM	31	65 (range 29–83)	21/10	9 (2–14)	LAR (23) APR (4) PC (3) PME (1)	NR	RT (4)
2004	Kneist et al. *[41]	Apr 2002–Oct 2002	Prospective	pIONM	17	61 (range 29–75)	11/6	2 (1–4)	LAR (14) PC (2) MVR (1)	NR	CRT (2)
2002	Hanna et al. [43]	NR	Prospective	pIONM	21	Mean 48.1 (range 28 -67)	21/0	6	LAR (21)	0/0/21	CRT (14)

*APR *abdominoperineal resection, *LAR *low anterior resection, *PC *proctocolectomy, *MVR *multi-visceral resection, *PC *proctocolectomy, *PME *partial mesorectal excision, *RR *resection rectopexy, *RPC *restorative proctocolectomy, *ISR *intersphincteric resection, *pIONM *intraoperative pelvic autonomic nerve monitoring, *PST *presacral tumour excision, *CRT *chemoradiotherapy, *RT *radiotherapy, *CT *chemotherapy, *taTME *transanal total mesorectal excision, *NR *not reported, *N/A *not applicable

*Denotes studies published from the same research group

^Denotes the same sample of patients studied at different follow−up periods

There was heterogeneity in the pIONM techniques and outcome measures (Table 2). The most common pIONM technique, reported in nine studies, used a laparoscopic bipolar microfork probe to perform electrical stimulation of pelvic autonomic nerves [18, 28, 30–35, 44]. Stimulation was performed bilaterally along the pelivic sidewall during surgery and immediately following resection of the specimen to stimulate the pelvic splanchnic nerves, S2–S4, and inferior hypogastric plexus. The functional integrity of the nerves was established based on the response to stimulation with intravesical pressure monitoring (IPM), where a positive stimulation induced detrusor muscle contraction and increased intravesical pressure and an increase in the electromyography amplitude recorded from a needle electrode in the internal anal sphincter (EMG of the IAS) [18, 28, 30–35, 44]. Three studies used the bipolar microfork technqiue but only observed EMG of the IAS [27, 29, 36]. Six studies used mixed techniques, including monopolar and bipolar stimulation, whilst only observing IPM [17, 37–41]. One study observed penile tumescence [43]. The results are described in terms of intact function bilaterally (both right and left pelvic sidewall stimulation producing a measurable effect), unilaterally (either right or left stimulation producing a measurable effect) or absent (no measured effect from any stimulation) [17, 18, 27, 29, 31, 32, 34–44]. There have been no reports of adverse events or complications related to pIONM.

**Table 2:** pIONM technical details and functional outcome measures

Author	pIONM technique	Observed response	Definition of functional integrity	Functional outcome measure										
				Anorectal function				Urinary function			Sexual function			QoL
				WS	DRESS	LARS Score	CCCS	IPSS	Residual urine volume	Urinary catheter requirement	IIEF	FSFI	Other	QoL Index for Urinary Symptoms
Jin et al. [27] (2020)	Bipolar laparoscopic probe Bilateral stimulation during dissection of the retrorectal space, lateral rectal ligament and Denonvilliers’ fascia. Stimulation again after specimen removed Electrical current range of 5–10 mA, frequency of 2 Hz and stimulation time of 5–20 s	EMG of IAS (needle electrode into IAS with reference electrode in left thigh)	Presence of evoked potentials in EMG of the IAS	X				X		X	X	X	*CIPE*	X
Zhou et al. [29] (2019)			NR	X				X			X	X		X
Kneist et al. [44] (2022)	Bipolar stimulation with a hand-guided microfork probe Bilateral posterolateral, lateral, anterior and at level of pelvic floor. During and after rectal surgery. Any macroscopic nerves also stimulated Electrical current of 6 mA, frequency of 30 Hz and monophasic rectangular pulses with pulse duration of 200 μs	EMG of IAS (needle electrode into IAS under endoanal ultrasound guidance with ground electrode in left gluteal muscle) Manometry of urinary bladder (bladder filled with 200 ml Ringer's solution and urinary catheter connected to pressure transducer linked to neuromonitoring device)	A stimulation-dependent unilateral or bilateral increase in intravesical pressure or EMG amplitude of the IAS	X				X	X	X	X	X		X
Kauff et al. [28] (2020)				X										
Kauff et al. [30] (2017)								X		X	X	X		X
Kauff et al. [18] (2016)				X	X			X		X	X	X		X
Kneist et al. [31] (2014a)				X	X			X			X			X
Kneist et al. [32] (2014b)				X	X		X	X	X		X	X		X
Kneist et al. [33] (2013a)				X	X			X	X	X	X	X		X
Kauff et al. [34] (2013)				X	X			X						X
Kneist et al. [35] (2013b)				X	X		X	X	X			X		X
Kneist et al. [42] (2016)	Bipolar stimulation with a hand-guided microfork probe Bilateral neuromapping performed during and after transabdominal and transanal mesorectal dissection Electrical current of 6–15 mA, frequency of 30 Hz and monophasic rectangular pulses with pulse duration of 200 μs			X		X		X	X		X	X		X
Kneist et al. [36] (2012)	Bipolar stimulation with a hand-guided microfork probe Bilateral posterolateral, lateral, anterior and at level of pelvic floor. During and after rectal surgery. Any macroscopic nerves also stimulated Electrical current of 6 mA, frequency of 30 Hz and monophasic rectangular pulses with pulse duration of 200 μs	EMG of IAS (needle electrode into IAS under endoanal ultrasound guidance with ground electrode in left gluteal muscle)	A stimulation-dependent unilateral or bilateral increase in EMG amplitude of the IAS	X	X									
Fang et al. [17] (2015)	Bilateral stimulation of splanchnic pelvic nerves arising from sacral roots sequentially carried out in region of laterodorsal pelvic wall after procedure Monopolar stimulation using constant voltage (Stimuplex HNS 12, B. Braun Melsungen AG, Melsungen, Germany) Current 3–5 mA, frequency of 2 Hz and stimulation lasting 5–20 s	Manometry of urinary bladder (bladder filled with 180–200 ml Ringer's solution and urinary catheter connected to manometer with an analogue scale)	An increase in intravesical pressure of > 1cmH20					X	X	X	X			X
Kneist et al. [37] (2007a)	Bilateral stimulation of splanchnic pelvic nerves arising from sacral roots sequentially carried out in region of laterodorsal pelvic wall after procedure Monopolar and bipolar stimulation using constant voltage (Screener 3625-Medtronic, Minneapolis, MN, USA; Vocare Surgical Stimulator-Neurocontrol, Cleveland, OH, USA) and constant-current stimulators (OSIRIS; inomed GmbH, Teningen, Germany) Current voltages 3–12 V, currents of 5–20 mA, frequency of 5–35 Hz and square pulses with pulse duration of 210–310 μs							X *	X	X				X
Kneist et al. [38] (2007b)								X *						X
Kneist et al. [39] (2007c)								X		X	X			
Kneist et al. [40] (2005)	Bilateral stimulation of splanchnic pelvic nerves arising from sacral roots sequentially carried out in region of laterodorsal pelvic wall after procedure Monopolar insulated rigid probe using a constant voltage stimulator (Screener 3625-Medtronic, Minneapolis, MN, USA) Current voltage 12 V, frequency of 30–35 Hz and rectangle impulses with pulse duration of 210–310 μs		An increase in intravesical pressure of > 2cmH20					X *	X	X				X
Kneist et al. [41] (2004)			A constant intravesical pressure rise					X *	X				*Non-validated questionnaire*	X
Hanna et al. [43] (2002)	Bilateral stimulation of the hypogastric nerves during dissection high on the pelvic sidewall Nerve stimulation probe (CaverMap® Device [Uromed Corp, Norwood, MA]) Stimulation protocol repeated after removal of the specimen	A tumescence sensor placed around the penis to measure changes in circumference	A change in tumescence of ‘+ 2’ recorded by CaverMap®										*Non-validated questionnaire*	
			Total	12	7	1	2	17	9	9	11	9	3	16
			Number of studies assessing functional outcome n (%)	12/20 (60%)				17/20 (85%)			14/20 (70%)			16/20 (80%)

*DRESS *digital rectal examination scoring system, *LAR S*low anterior resection syndrome, *CCCS*C leveland Clinic Constipation Score, *IPSS *International Prostate Symptom Score, *IIEF *International Index of Erectile Function, *FSFI *Female Sexual Function Index, *QoL *quality of life, *EMG *electromyography, *IAS *internal anal sphincter, *CIPE *Chinese Index of Premature Ejaculation, *WS *Wexner score

*Denotes women were asked additional questions to assess urinary incontinence

Earlier reports describe pIONM as a diagnostic tool to identify patients at risk of postoperative functional sequelae [37, 40, 41], making it possible to establish the diagnostic accuracy of pIONM from five studies [18, 31, 32, 34, 36]. These studies originated from two authors; however, the risk of patients being reported twice was considered small. In the two reports by Kauff et al., one manuscript was submitted for publication in February 2013 [34] whilst the later manuscript reports a study period starting in May 2013 [18]. In those published by Kneist et al., the authors report demographic and clinical data including age, gender, tumour location, stage and the use of neoadjuvant therapy. By comparing these data between reports, it is possible that only 1 patient from an earlier study of 14 patients [36] could be reported in a later study of 17 patients [31]. A further patient from this later study may have been reported in a series of 10 patients by the same author [32] leaving data from only two patients which may be reported twice.

Recently, studies have described pIONM as a tool to identify and therefore protect pelvic autonomic nerves from injury with results presented here narratively [17, 28–30, 44].

The risk of bias assessments using the QUADS-2 tool are displayed in Table 3. All but four studies were at risk of bias whilst only eight studies demonstrated concerns regarding applicability.

**Table 3:**
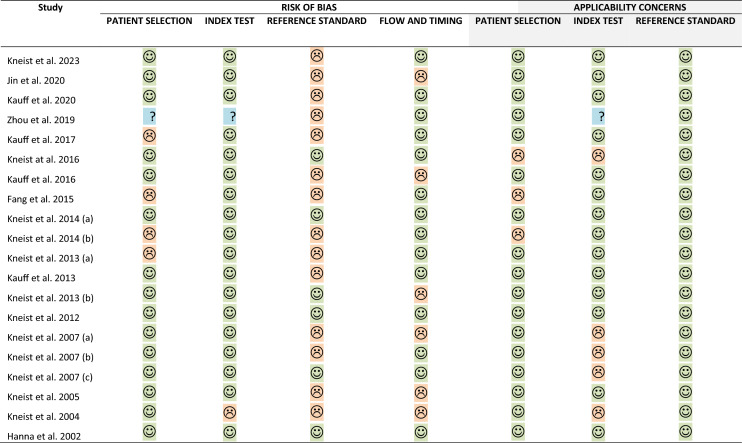
Risk of bias assessements using the QUADS-2 tool

### Functional outcomes

#### Anorectal function

Anorectal function was assessed in 12 of the 20 included studies with the Wexner score (WS) being the reported outcome in all [18, 27–29, 31–36, 42, 44]. The WS is a validated patient-reported faecal incontinence symptom severity measure with scores ranging from 0, indicating no symptoms, to 20 indicating severe symptoms of faecal incontinence [45]. One study reported the LARS score [42].

##### Diagnostic accuracy of pIONM

**Fig. 2:**
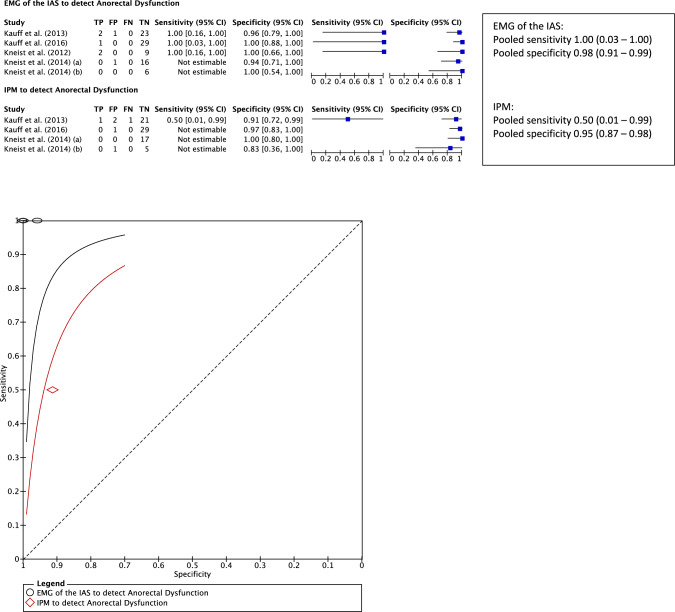
Summary receiver-operator curve and forest plot demonstrating the sensitivity and specificity of a bilaterally absent pIONM stimulation response in electromyography of the internal anal sphincter or intravesical pressure monitoring in the detection of new anorectal dysfunction defined as a Wexner score > 9. New anorectal dysfunction defined as a postoperative deterioration in the Wexner score to > 9. An absent response to bilateral stimulation of the pelvic autonomic nerves indicates a negative pIONM result. Either bilateral or unilateral positive response to stimulation indicates a positive pIONM result. In the forest plots the sensitivity and specificity for detecting anorectal dysfunction in each individual study are reflected by the blue squares with the 95% confidence intervals represented by the black lines. *pIONM* pelvic autonomic nerve monitoring, *EMG* electromyography, *IAS* internal anal sphincter, *IPM* intravescical pressure monitoring

A new postoperative WS of > 9 was used to define AD [18, 28, 33, 34]. Using this definition, the diagnostic accuracy of a bilaterally absent pIONM result, indicating bilateral impaired nerve function, to detect new postoperative AD can be established. Data from five studies with the same pIONM technique were used to calculate the diagnostic accuracy of pIONM [18, 31, 32, 34, 36] (Fig. 2). Four patients were excluded from one study (resection rectopexy = 2, abdominoperineal excision = 2) [32]. In four studies [18, 31, 32, 34], both IPM and EMG of the IAS were observed, and in one study only EMG of the IAS was observed [36]. The overall incidence of new AD was low (*n* = 5/90, 5.6%). A bilaterally absent response observed in EMG of the IAS showed a greater pooled sensitivity across three studies (1.00, 95% CI 0.03–1.00) compared to IPM in one study (0.50, 95% CI 0.01–0.99). These results and wide 95% confidence intervals are influenced by the low numbers of true-positive or false-negative cases with all studies reporting a sensitivity of 1.00 in detecting AD with EMG of the IAS, for example. Specificity was greater in all five studies measuring EMG of the IAS (0.98, 95% CI 0.91–0.99) compared to all four studies observing IPM (0.95, 95% CI 0.87–0.98). This is reflected in the ROC curves where the curve representing EMG of the IAS sits closer to the point of perfect classification at the top of the y-axis (Fig. 2).

##### Clinical utility of pIONM

In studies where it was not possible to calculate the diagnostic accuracy of pIONM, it was considered an intervention to reduce the risk of postoperative functional complications. In one study, non-performance of pIONM was identified as an independent predictor of AD after TME at 24-month follow-up (pIONM: 6/28, 21.4%. no pIONM: 11/22, 50.0%, *p* = 0.0035) [28]. In the only included randomised controlled trial, the WS was higher, indicating worse symptoms of faecal incontinence at 12-month follow-up in the control group (mean [standard deviation] 7.9 [5.6] vs 5.5 [4.5], *p* = 0.011) [44]. Jin et al. [27] and Zhou et al. [29] also report a higher WS in controls (6.00 [1.70] and 8.48 [3.34]) compared to patients undergoing pIONM with unilaterally intact (4.90 [0.88] and 8.10 [2.60]) or bilaterally intact (4.21 [1.47] and 6.85 [2.24]) results.

#### Urinary function

Seventeen studies assessed urinary function, and all used the International Prostate Symptom Score (IPSS) [17, 18, 27, 29–35, 37–44] with only three of these asking female patients additional questions to assess symptoms of stress urinary incontinence or dysuria, but these were non-validated tools [37, 38, 40]. The remaining studies that included female patients did not report a validated questionnaire for female urinary function. The IPSS is a symptom severity questionnaire initially validated to quantify symptoms of urinary dysfunction in male patients with benign prostatic hyperplasia. The results of the seven-domain questionnaire range from 0, indicating no urinary dysfunction, to 35, suggesting symptoms of severe dysfunction [46]. The QoL index for urinary symptoms, included as an additional part of the IPSS, was reported in 16 of the studies that assessed urinary function [17, 18, 27, 29–35, 37, 38, 40–42, 44]. Other measures included an excess residual urinary volume (> 100 ml) in nine studies [17, 32, 33, 35, 37, 40–42, 44] or the need for ongoing urinary catheterisation in nine studies [17, 18, 27, 30, 33, 37, 39, 40, 44].

##### Diagnostic accuracy of pIONM

**Fig. 3:**
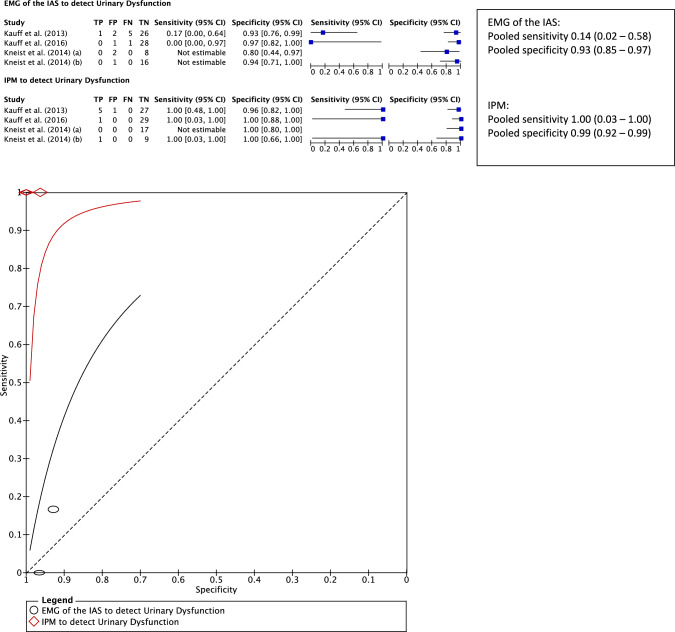
Summary receiver-operator curve and forest plot demonstrating the sensitivity and specificity of a bilaterally absent pIONM stimulation response in electromyography of the internal anal sphincter or intravesical pressure monitoring in the detection of new urinary dysfunction defined as a deterioration in IPSS and QoL index for urinary symptoms or the need for ongoing urinary catheterisation. New urinary dysfunction is defined as a deterioration in IPSS and QoL index for urinary symptoms or the need for ongoing urinary catheterisation. An absent response to bilateral stimulation of the pelvic autonomic nerves indicates a negative pIONM result. Either bilateral or unilateral positive response to stimulation indicates a positive pIONM result. In the forest plots the sensitivity and specificity for detecting urinary dysfunction in each individual study are reflected by the blue squares with the 95% confidence intervals represented by the black lines. *pIONM* pelvic autonomic nerve monitoring, *IPSS* International Prostate Symptom Score, *QoL* quality of life, *EMG* electromyography, *IAS* internal anal sphincter, *IPM* intravescical pressure monitoring

New UD was defined as a deterioration in the IPSS and QoL index (increasing score) or the need for ongoing urinary catheterisation [17, 18, 27, 29–35, 37, 38, 40–42]. In studies with the same pIONM technique, the diagnostic accuracy of a bilaterally absent pIONM result in detecting UD can be established in four studies of patients undergoing TME [18, 31, 32, 34] (Fig. 3). Three of these included female patients [18, 32, 34]. As with AD, the incidence of UD is low (*n* = 7/91, 7.7%); however, in contrast to AD, a bilaterally absent response observed with IPM is superior to EMG of the IAS in detecting UD with a higher pooled sensitivity (1.0, 95% CI 0.03–1.00 vs 0.14, 95% CI 0.02–0.58) and specificity (0.99, 95% CI 0.92–0.99 vs 0.93, 95% CI 0.85–0.97). The wide 95% confidence intervals of pooled sensitivity results are again influenced by the low number of true-positive and false-negative cases, with all studies reporting a sensitivity of 1.00 in detecting UD with IPM. These results are represented by the position of the ROC curve for IPM sitting closer to the point of perfect classification (Fig. 3).

##### Clinical utility of pIONM

Where the diagnostic accuracy of pIONM could not be established the technique was again reported as an intervention to reduce the incidence of UD. Urinary function measured at 12 months postoperatively in the NEUROS randomised controlled trial deteriorated more frequently in the control group (OR: 0.343, 95% CI 0.124–0.944, *p* = 0.038) [44]. The non-performance of pIONM was identified as an independent predictor of UD after TME in one study at 24-month follow-up (pIONM: 8/40, 20.0%. no pIONM: 20/39, 51.3%, *p* = 0.004)[30]. Using EMG of the IAS as the only observed response, Jin et al. and Zhou et al. reported higher IPSS, indicating worse urinary function, in controls (5.19 [3.30] and 9.10 [4.08]) compared to patients with unilaterally intact (3.00 [1.33] and 10.23 [3.09]) or bilaterally intact (2.94 [2.49] and 6.76 [2.53]) pIONM results [27, 29].

#### Sexual function

Sexual function was assessed in 14 of the 20 included studies with the International Index of Erectile Function (IIEF) and the Female Sexual Function Index (FSFI) most frequently used to assess males and females, respectively [17, 18, 27, 29, 30, 32, 33, 35, 39, 41–44].

##### Diagnostic accuracy of pIONM

It was not possible to establish the diagnostic accuracy of pIONM with the data available.

##### Clinical utility of pIONM

The non-performance of pIONM was identified as an independent predictor of new SD (reduced IIEF or an FSFI < 26.55) after TME in one study at 24-month follow-up (pIONM: 14/25, 56.0%. no pIONM: 19/21, 90.5%, *p* = 0.010) [30]. This is in contrast to results of the randomised controlled trial which found no difference in the incidence of SD in either the control or pIONM groups, although this was felt to be influenced by the high baseline SD in both groups [44].

## Discussion

This systematic review aimed to assess the diagnostic accuracy of pIONM in the identification of postoperative functional complications in patients undergoing rectal surgery. It indicates that pIONM may have potential in identifying these functional complications but both EMG of the IAS and IPM should be used for maximum diagnostic accuracy of AD and UD, respectively (Figs. 2 and 3). Given the difficulties in identifying pelvic nerves, pIONM was suggested as an alternative to visual identification alone [32, 47]. Initial reports highlighted pIONM as a diagnostic test to identify patients at risk of postoperative functional complications which should prompt early and proactive intervention to ameliorate symptoms [38, 41]. However, more recent studies have described pIONM as an intervention used to reduce the risk of these complications which limited the number of studies to establish the diagnostic accuracy of pIONM [28, 30, 44]. This apparent benefit as a tool for primary prevention of postoperative dysfunction may be due to an improved ability to identify pelvic nerves that are at risk of damage and change the surgical strategy [47, 48].

This review found heterogeneity in the reported techniques of pIONM and patient-reported outcomes measures used across the literature. Bipolar electrical stimulation is the preferred technique as it provides a concentrated electrical current between the electrodes [37]. In the assessment of AD, all included studies used the Wexner score to measure the severity of faecal incontinence. However, only one included study used the LARS score in patients undergoing transanal TME [42]. Several factors may contribute to the development of LARS, including motor and sensory denervation of the rectum and anal canal and loss of anorectal reflexes [49]. LARS remains a feared complication following rectal resection with a profound negative impact on QoL due to disordered bowel function after rectal resection [50]. The LARS score has shown a high diagnostic sensitivity (72.5%) and specificity (82.5%) as well as a correlation with QoL [51]. The NEUROS randomised trial identified fragmented defecation was less frequent in the pIONM group (56% vs 75%) highlighting the potential of pIONM in the prevention of LARS [44]. Although QoL after rectal cancer surgery typically returns to baseline after 12 months [52, 53], no study in this review examined overall or symptom-specific QoL. Future work with pIONM should look to include an assessment of LARS and QoL, which are important omissions in the included studies.

Rectal surgery requires a detailed understanding of complex neuroanatomy to protect pelvic nerves. Several areas have been highlighted where they are at risk of iatrogenic injury including ligation of the inferior mesenteric artery, dissection of the lateral ligaments, Denonvilliers’ fascia and the retrorectal space [54, 55]. Even when the pelvic nerves are identified, their functional integrity cannot be confirmed with visualisation alone [18]. Iatrogenic injury to pelvic nerves and use of neoadjuvant radiotherapy have been identified as risk factors for long-term UD after TME in up to 38% of cases [56, 57]. pIONM may be a useful tool for detecting UD and its omission has also been associated with an increased risk of developing UD [30, 44]. Postoperative SD is common and constitutes a diverse spectrum of symptoms with a prevalence of up to 70% [52, 58]. In common with AD and UD, neoadjuvant radiotherapy increases the risk of postoperative SD [4, 58]. The use of pIONM in non-randomised studies led to a reduced incidence of SD in the pIONM group [27, 29, 30].

This systematic review offers clinically relevant diagnostic accuracy results on the potential of pIONM to detect postoperative AD and UD. It also highlights how using pIONM could reduce the incidence of functional sequelae by supporting the surgeon to identify and protect pelvic autonomic nerves. There are however limitations to this review including the presence of only one randomised trial, the relatively small sample sizes, short follow-up periods and variations in pIONM techniques and outcome measures. The low prevalence of dysfunction has led to wide confidence intervals of pooled sensitivity and specificity results. In addition, many of the reported studies came from the same research group, which may limit the generalisability of the findings. The NEUROS study attempted to address this by involving additional surgical units who received training in the pIONM technique. Finally, using questionnaires as an instrument to detect functional sequelae relies on a patient’s assessment of their symptoms, and various methods have been used to confirm the validity of each questionnaire as a diagnostic tool [59].

## Conclusions

This review suggests pIONM has potential in detecting postoperative functional complications following rectal surgery. There is a need to standardise the technique and outcome measures whilst generating more robust data through larger randomised trials that adjust for all other confounding variables. Use of pIONM could be considered as an adjunct in rectal surgery to reduce the risk of nerve injury, particularly in patients at high risk of functional complications.

## Data Availability

No datasets were generated or analysed during the current study.
